# A new ELISA kit which uses a combination of *Plasmodium falciparum *extract and recombinant *Plasmodium vivax *antigens as an alternative to IFAT for detection of malaria antibodies

**DOI:** 10.1186/1475-2875-6-19

**Published:** 2007-02-21

**Authors:** Cecile Doderer, Aurelie Heschung, Phillippe Guntz, Jean-Pierre Cazenave, Yves Hansmann, Alexandre Senegas, Alexander W Pfaff, Tamer Abdelrahman, Ermanno Candolfi

**Affiliations:** 1 Institut de Parasitologie et de Pathologie Tropicale, 3 rue Koeberlé, 67000 Strasbourg cedex, France; 2 Etablissement Français du Sang d'Alsace, 10 rue Spielmann, 67000 Strasbourg cedex, France; 3 Service de Maladies Infectieuses et Tropicales, Hôpitaux Universitaires de Strasbourg, 1 place de l'Hôpital, 67000 Strasbourg cedex, France

## Abstract

**Background:**

The methods most commonly used to measure malarial antibody titres are the Indirect Fluorescence Antibody Test (IFAT), regarded as the gold standard, and the Enzyme-Linked ImmunoSorbent Assay (ELISA). The objective here was to assess the diagnostic performance, i.e. the sensitivity and specificity, of a new malaria antibody ELISA kit in comparison to IFAT. This new ELISA kit, the ELISA malaria antibody test (DiaMed), uses a combination of crude soluble *Plasmodium falciparum *extract and recombinant *Plasmodium vivax *antigens.

**Methods:**

Two groups were used: 95 samples from malaria patients to assess the clinical sensitivity and 2,152 samples from blood donors, who had not been exposed to malaria, to assess the clinical specificity.

**Results:**

The DiaMed ELISA test kit had a clinical sensitivity of 84.2% and a clinical specificity of 99.6% as compared with 70.5% and 99.6% respectively, using the IFAT method. The ELISA method was more sensitive than the IFAT method for *P. vivax *infections (75% *vs*. 25%). However, in 923 malaria risk donors the analytical sensitivity of the ELISA test was 40% and its specificity 98.3%, performances impaired by large numbers of equivocal results non-concordant between ELISA and IFAT. When the overall analytical performances of ELISA was compared to IFAT, the ELISA efficiency *J *index was 0.84 versus 0.71 for IFAT. Overall analytical sensitivity was 93.1% and the analytical specificity 96.7%. Overall agreement between the two methods reached 0.97 with a reliability *k *index of 0.64.

**Conclusion:**

The DiaMed ELISA test kit shows a good correlation with IFAT for analytical and clinical parameters. It may be an interesting method to replace the IFAT especially in blood banks, but further extensive investigations are needed to examine the analytical performance of the assay, especially in a blood bank setting.

## Background

More than 2 billion people (40% of the world's population) live in areas where malaria is endemic. It was estimated that over 250 million people worldwide contracted malaria in 2002 [1]. Following infection with any of the four species of *Plasmodium*, specific antibodies are produced, in virtually all individuals, one or two weeks after initial infection and persist for three to six months after parasite clearance. These antibodies may persist for months or years in semi-immune patients in endemic countries where reinfection is frequent. However, in a non-immune patient, treated for a single infection, antibody levels fall more rapidly and may be undetectable by three to six months. Reinfection or relapse leads to a secondary response with a high and rapid rise in antibody titres [2,3]. Antibody detection is definitely not a substitute for blood film examination in the diagnosis of an acute attack of malaria, and is mainly used in screening of prospective blood donors to avoid transfusion-transmitted malaria [4,5]. Nowadays, that risk is still high due to the extensive exchanges between malaria endemic areas and non-endemic areas [4,6]. Malaria occurring in travellers to the tropics is mainly due to *Plasmodium falciparum *(60%) and *Plasmodium vivax *(24%) [7]. Anti-malarial antibodies can be detected by various methods, which are, however, believed to lack both sensitivity and specificity [8]. Immuno-Fluorescence Antibody Test (IFAT) is still regarded as the gold standard for malarial serology and until recently was the only validated method for detecting *Plasmodium*-specific antibodies in blood banks [9]. IFAT is a simple and sensitive method, but it is time-consuming and difficult to automate. It requires fluorescence microscopy and trained technicians, making it operator-dependent and subjective, particularly for serum samples with low antibody titres. Additionally, the lack of standardization of IFAT reagents and manipulations makes it impossible to harmonize inter-laboratory results. Moreover, the antigen is obtained by *in vitro *culture of *P. falciparum *and gives very good sensitivity for this species, but shows limited cross reactivity with other human pathogenic species. An interesting solution would be to add an IFAT technique with *Plasmodium cynomolgi *antigens to detect anti-*P. vivax *antibodies, but this would be impossible to apply routinely in blood transfusion centres [10,11]. More reproducible and easy to automate, ELISA methods, using crude soluble antigen, lack sensitivity compared to IFAT [12-14] but the more recent arrival of enzyme immunoassays using recombinant antigens [15] has provided a more sensitive and practical alternative to IFAT.

Here a new ELISA kit (ELISA malaria antibody test, DiaMed, Switzerland) was evaluated, which combines soluble *P. falciparum *antigens and recombinant *P. vivax *antigens and detects both IgG and IgM. This kit was compared with the IFAT method routinely used. First, the sensitivity of the two methods was determined with samples from patients with clinical signs of malaria, using direct examination as the reference method, in the knowledge that anti-malarial antibodies are produced virtually in all subjects one or two weeks after initial infection with all four species and persist for 3–6 months after parasite clearance [13,16]. Then the specificity of the two methods was determined by testing a panel of sera from blood donors not exposed to malaria and from malaria-risk donors.

## Materials and methods

### Samples from *Plasmodium *infected patients

Sera from 95 patients were used to compare the performance of ELISA and IFAT. Seventy-six patients had returned with fever from an endemic country and were microscopically diagnosed positive for *P. falciparum *(n = 66), *P. vivax *(n = 5), *Plasmodium ovale *(n = 2), *Plasmodium malariae *(n = 2), or mixed *P. falciparum *and *P. malariae *infection (n = 1). These patients completed a questionnaire on endemic zones visited and were divided into two categories: i) patients returning from their first trip to a malaria-endemic area and considered as having a primary infection (n = 32), and ii) patients having visited endemic areas several times and considered as frequent tropical travellers and possibly reinfected (n = 44). Nineteen patients were infected by *P. vivax *in Venezuela and were considered as residents (sera kindly provided by Dr. Hilda A. Perez, Laboratorio de Immunoparasitologia of the Venezuelan Institute of Scientific Research, Caracas, Venezuela). These samples were used to calculate the clinical sensitivity.

### Samples from blood donors

Sera from blood donors were collected at Etablissement Français du Sang d'Alsace (EFS Alsace). On the basis of a medical questionnaire, donors were selected and classified as "not-exposed-to-malaria blood donors" (2152 sera) if they had not travelled to malarial endemic areas in the last three years, the group that was used for the calculation of the clinical specificity, and as "malaria-risk blood donors" (923 sera) if they had returned from a malarial endemic area between the last four months to three years. This last group of malaria-risk blood donors was used to calculate the analytical sensitivity and specificity.

### IFAT method

IFAT was employed as the reference method. Ready-to-use slides bearing spots coated with *P. falciparum *antigen were used. The antigen was prepared from A1 human red blood cell cultures *in vitro *(Falciparum-Spot IF, bioMérieux, Marcy l'Etoile, France). Sera were diluted 1/30 and 1/60 in PBS before testing. Ten μL of each dilution were applied to a spot, and the slides were incubated for 25 minutes at 37°C. One positive control and one negative control were included in each series. The slides were washed twice for 10 minutes in PBS, then rinsed and dried before adding fluorescein-labelled total human anti-Ig conjugate (bioMérieux, Marcy l'Etoile, France) diluted at 1/200 in PBS containing Evans blue (0.1 g/L). After incubation at 37°C for 25 minutes the slides were again washed twice for 10 minutes in PBS, then rinsed and dried. The slides were read under a fluorescence microscope (Zeiss) with UV illumination and a × 40 objective. Samples fluorescing at 1/30 dilution were considered equivocal, and samples fluorescing at 1/60 dilution were considered positive following the in-house validated protocol [17-19].

### ELISA malaria antibody test

The DiaMed ELISA malaria antibody test is based on binding of anti-*Plasmodium *antibodies present in a serum sample to antigens immobilized on 96-well plates. The antigens are a mixture of a total extract of cultured *P. falciparum *and recombinant *P. vivax *antigens (MSP1 and CSP). The test was done as recommended by the manufacturer, as follows. Ready-to-use diluent buffer (125 μL) was dispensed into each well, followed by 25 μL of test serum. On the same plate, 25 μL positive control and negative control were also dispensed in single well and triplicate wells respectively. The plate was then covered and incubated for 60 minutes at 37°C before being washed 5 times. Horseradish peroxidase-conjugated rabbit anti-human IgM and IgG monoclonal antibodies (100 μL) were added to each well and the plates were incubated for 30 minutes at 37°C. The wells were again washed 5 times, and 100 μL of substrate solution were added to each well. The plate was covered and incubated in the dark for 15 minutes at 37°C. Finally, 50 μL of 0.5 M sulphuric acid was added to each well and absorbance was read within 15 minutes at 450 nm, with a reference wave-length of 620 nm. The cut-off value was calculated by multiplying the average optical density (OD) of the negative controls wells by four (with a minimum value of 0.200). The antibody (Ab) index of each determination is obtained by dividing the OD value of each sample by the cut-off value. A negative reaction corresponds to an Ab index of 0.8 or less, a positive reaction to an Ab index of 1.0 or more, and an equivocal result to an Ab Index between 0.8 and 1.0. The test does not distinguish between IgG and IgM, or between antibodies to *P. falciparum*, *P. vivax*, *P. ovale *and *P. malariae*.

This assay was carried out manually at the IPPTS, but was adapted to automates for sample distribution (Genesis-Tecan and Microlab AT 2+ from Hamilton) and microplate managers (Ortho Summit Processor from Ortho Clinical Diagnostics and BEP III from Dade Behring) at EFS Alsace for the specificity study.

### Investigations

All samples were tested using both IFAT and ELISA. IFAT, for all samples, was performed at the IPPTS. ELISA, except in the case of clinical samples, was performed at the EFS Alsace.

### Statistical analysis

Fisher's exact test was used to compare the performances of the assays. Sensitivity, specificity, 95% confidence interval (CI) and Youden's efficiency index (*J*) were calculated for both assays. Concordance and Cohen's Kappa inter-rater reliability index (*k*) were also calculated between the two tests. *J *and *k *indexes have a range from 0–1.00, with larger values indicating better efficiency and reliability. Equivocal results for the same sera with both assays were excluded from the calculations. Equivocal results were considered as negative for sensitivity calculation, as positive for specificity calculation and for global analysis. All equivocal cases are clearly identified in the results section.

## Results

### Malaria-infected patients

In the samples from *Plasmodium-*infected patients, the IFAT and ELISA sensitivity was 70.5% (67/95) and 84.2% (80/95) respectively so not significantly different. Correlation between the two methods was 0.76. More equivocal results were observed with IFAT compared to ELISA (6 *vs*3). The mean and SD of ELISA ratios for the malaria-infected group was 2.05 ± 1.6 (Table 1). These differences may be may be explained by the detection of *P. vivax *cases. Indeed, the performance of the IFAT and ELISA tests varied according to the species (Table 2); the ELISA method was three times more sensitive for *P. vivax *infection (24 cases, 75% for ELISA and 25% for IFAT). Both tests failed to detect primary malarial infections. Considering the cases diagnosed in Strasbourg, IFAT and ELISA both failed to detect three cases of *P. falciparum *from three non-immune patients, considered as first-time tropical travellers who had returned from West Africa less than three months before (respectively three days, 10 days and two weeks). For the nine *P. vivax *cases diagnosed at IPPTS, three cases were not detected by ELISA and IFAT and three by IFAT only. The three cases not detected by both ELISA and IFAT were from first-time tropical travellers and *P. vivax *infection was microscopically diagnosed two months, three months and 12 months after their return to Europe. For the three remaining *P. vivax *cases, detected by ELISA and not detected by IFAT, all were also from first-time tropical travellers more three months after their return from West Africa (four, five and 11 months). For the 19 Venezuelan *P. vivax *cases, considered as residents, three were missed by ELISA and IFAT, and nine by IFAT only. Next, it was asked whether there are any differences between first-time tropical travellers and multiple-time tropical travellers or patients resident in malarial areas for malarial antibody detection. Clinical sensitivity performances are similar for both methods for primary-infected patients compared to semi-immune patients (Table 3), except for *P. vivax *cases, as previously stated. Concordance for first-time travellers and frequent travellers were respectively 0.81 and 0.84. Of note and not surprisingly, a positive serology was more often observed for frequent travellers or malaria-area residents, as compared with primary infections.

**Table 1:** ELISA and IFAT comparative results for *Plasmodium *infected patients

		**ELISA malaria antibody test**			
		
		**Positive**	**Equivocal**	**Negative**	***Total***
**IFAT**	**Positive**	64	3	0	*67*
	**Equivocal**	3	0	3	6
	**Negative**	13	0	9	*22*
	***Total***	*80*	*3*	*12*	*95*

IFAT and ELISA clinical sensitivities respectively 70.5% (67/95) (CI: 59.6% – 81%) and 84.2% (80/95) (CI: 76.2% – 92.2%), equivocal results considered as negative for calculation.

**Table 2:** Positive results of ELISA and IFAT rates according to infecting *Plasmodium *species

**Species (# cases; %)**	**ELISA**	**IFAT**
*P. falciparum *(66 cases; 69.5%)	95.5% 63/66	95.5% 63/66
*P. vivax *(24 cases, 21%)	75% 18/24	25% 6/24
*P. ovale *(2 cases; 2.1%)	100% 2/2	100% 2/2
*P. malariae *(2 cases; 2.1%)	100% 2/2	100% 2/2
Mixed (1 case * ; 1%)	100% 1/1	100% 1/1

* P. falciparum plus *P. malariae*

**Table 3:** ELISA and IFAT results for first-time travellers (A) and frequent travellers or malaria area residents (B)

**A**		**ELISA malaria antibody Test**			
		
		**Positive**	**Equivocal**	**Negative**	***Total***
**IFAT**	**Positive**	20	1	0	*21*
	**Equivocal**	1	0	1	*2*
	**Negative**	3	0	6	*9*
	***Total***	*24*	*1*	*7*	*32*

IFAT and ELISA clinical sensitivities are respectively 65.6% (21/32) (CI: 45.3 – 85.9%) and 75% (24/32) (CI: 57.7 – 92.3%), equivocal results considered as negative for calculation.					

**B**		**ELISA malaria antibody test**			
		
		**Positive**	**Equivocal**	**Negative**	***Total***

**IFAT**	**Positive**	37	2	0	*39*
	**Equivocal**	2	0	2	*4*
	**Negative**	1	0	0	*1*
	***Total***	*40*	*2*	*2*	*44*

IFAT and ELISA clinical sensitivities are respectively 88.6% (39/44) (CI: 78.7 – 98.6%) and 90.9% (40/44) (CI/82 – 99.8%), equivocal results considered as negative for calculation.					

### Not-exposed-to-malaria blood donors

The clinical specificity for routine healthy non-exposed-to-malaria blood donors (2152) was 99.6% for both methods and raised concordance to 99.3% (Table 4). In both methods, there were seven false positives but none of them were concordant between the two methods, the difference being not significant. The mean ELISA Ab index for all the blood donors samples was 0.279 with an SD of 0.172 (median = 0.241), having in mind that the grey zone starts at 0.800 and results are positive with a ratio over or equal to 1.000. No other information was available on the subjects presenting positive or equivocal results for IFAT and/or ELISA. At that stage, the global clinical performances of both methods, in *Plasmodium*-infected patients and not-exposed-to-malaria blood donors, were statistically similar (P = 0.331).

**Table 4:** ELISA and IFAT results for not-exposed-to-malaria blood donors

		**ELISA malaria antibody test**			
		
		**Positive**	**Equivocal**	**Negative**	***Total***
IFAT	**Positive**	0	0	4	*4*
	**Equivocal**	0	0	3	*3*
	**Negative**	6	1	2138	*2145*
	***Total***	*6*	*1*	*2145*	*2152*

IFAT and ELISA clinical specificities: 99.6% (2145/2152) (CI: 99.4 – 99.9%), equivocal results considered as positive for calculation.

### Malaria-risk blood donors

When tested, the 923 serum samples from healthy malaria-risk blood donors (Table 5), four samples were positive using both the IFAT and ELISA methods, and of the 908 serum samples found negative by IFAT, 888 were also negative by ELISA, with a ELISA Ab index mean and SD of 0.380 ± 0.270. 31 ELISA false-positive results were observed as compared to IFAT, the specificity falling to 96.8% (879/908). Among them 14 were equivocal for ELISA and not confirmed by IFAT. However, it had previously been observed in the *Plasmodium*-infected patients that the performance of ELISA was better for *P. vivax *cases, and it was suggested that this may be due to the presence of antibodies directed to *P. vivax*. Consequently, the 31 sera negative by IFAT and positive/equivocal by ELISA were sent to the French National Reference Center for Malaria (Prof. J. Le Bras, Laboratoire de Parasitologie, Hôpital Bichat-Claude Bernard, Paris) for an IFAT using *P. cynomolgi *antigens, the simian parasite *P. cynomolgi *from rhesus monkeys being an alternative source of antigen for serodiagnosis of *P. vivax *infection. All IFAT-*P. cynomolgi *returned negative. Similarly, the ELISA analytical sensitivity was poor, falling to 40%, with a really poor 95% CI, essentially due to sera equivocal for IFAT and negative for ELISA.

**Table 5:** ELISA and IFAT results for malaria-risk blood donors

		**ELISA malaria antibody test**			
		
		**Positive**	**Equivocal**	**Negative**	***Total***
**IFAT**	**Positive**	4	0	0	*4*
	**Equivocal**	2	0	9	*11*
	**Negative**	15	14	879	*908*
	***Total***	*21*	*14*	*888*	*923*

ELISA analytical specificity and sensitivity are respectively 96.8% (879/908) (CI: 95.6 – 98%) and 40% (6/15) (CI: 0.8 – 79.2%) in comparison to IFAT, equivocal results considered as positive for calculation.

### Overall clinical performances

These showed an efficiency *J *index of 0.71 for IFAT and of 0.84 for ELISA, if the diagnosis of malaria is considered as the reference (95 cases of microscopically diagnosed malaria, irrespective of the species and 3075 cases of healthy subjects without any clinical signs of malaria). The comparison of the global performances of ELISA versus IFAT showed that the concordance was 0.97 and the reliability *k *index 0.64 (Table 6).

**Table 6:** ELISA and IFAT results for all subjects

		**ELISA malaria antibody test**			
		
		**Positive**	**Equivocal**	**Negative**	***Total***
**IFAT**	**Positive**	68	3	4	*75*
	**Equivocal**	5	0	15	*20*
	**Negative**	34	15	3026	*3075*
	***Total***	*107*	*18*	*3045*	*3170*

ELISA analytical sensitivity and specificity in comparison to IFAT are respectively 90.6% (68/75) (CI: 83.8 – 97.6%) and 98.7% (3056/3095) (CI: 98.3 – 99.1%), equivocal results considered as negative.

## Discussion and conclusion

*Plasmodium *infection triggers the synthesis of specific antibodies, especially of the IgM and IgG isotypes (IgA levels also rise, but to a lesser extent). Antibodies to all four *Plasmodium *species are produced by virtually all individuals 1–14 days after infection [13,16]. Antibody titres decline rapidly following recovery from primary infection, and disappear within about a year. The presence of specific antibodies is thus a marker of recent contact with *Plasmodium*. Specific antibody titres are proportional to the intensity and duration of infection, and serological methods are more sensitive than direct examination, when used to assess past or present malaria infection, except in the acute stage of the first infection, as the antibody titre is independent of the date of infection and fluctuations in blood parasite levels [2,3,20].

Serological methods have three main uses: a) screening in transfusion centres located in non-endemic zones, with large numbers of donors returning from endemic regions; b) diagnosis of fever of unknown origin, especially in patients inadequately treated for malaria and patients with tropical splenomegaly: the mean antibody titre reflects the intensity of contact with the parasite; and c) epidemiological studies [11]. IFAT is considered as the gold standard, but it is time consuming and lacks reproducibility. Is a newly-established malaria ELISA antibody test, combining native and recombinant-detecting antigens, able to replace IFAT?

The ELISA performance in acute parasitaemic malaria infection provides a way to measure its ability to detect potential asymptomatic but parasitaemic patients. In the present study, the DiaMed ELISA malaria antibody test had an overall sensitivity of 84.2% in patients with malaria, compared to 70.5% with the IFAT method. Both ELISA and IFAT methods still miss some cases of acute infection, owing to the immunological window between infection and antibody production, and to the high variability of *Plasmodium *blood-stage antigens. Closing the window by the use of antigen detection or PCR are under investigation [21]. For *P. falciparum *cases, ELISA performances were similar to IFAT and markedly different for *P. vivax *cases.

First generation ELISA kits, based exclusively on *P. falciparum *antigens, showed a poor sensitivity for *P. vivax *infection [22-24]. The relatively poor sensitivity of other ELISA methods could be due to the use of soluble *P. falciparum *antigens without *P. vivax *antigens [13]. That point reminds that serology remain very-species specific with a substantial loss of titre when the right species is not used. This is due to poor cross-reactivity among *Plasmodium *species. The use of only one *Plasmodium *species for serological diagnosis is theorically inadequate. The use of at least three different antigens (*P. falciparum*, *P. vivax*, *P. malariae*) should be used. Practically, since Trager and Jensen continuous *in vitro *culture of *P. falciparum *discovery in 1976, only *P. falciparum *was used in laboratories. *P. vivax *antigens are not available on an industrial scale, as *P. vivax *cannot be cultured for long periods. The use of simian parasite such as *P. cynomolgi *for *P. vivax *or *P. brasilianum *for *P. malariae *is of limited use and is to be avoided for large-scale use. The advent of recombinant technology has allowed for diagnostic purposes the production of recombinant antigens from *P. vivax*. [25,26]. The performance of an assay, using four recombinant antigens of *P. falciparum *and *P. vivax *(The Newmarket Malarial Ab EIA from Newmarket laboratories, Newmarket, England), as well as the data presented here confirm that point [15,27]. Despite of the low numbers of cases in both studies, these data clearly suggest that the use of *P. vivax *recombinant antigens in a malaria antibody ELISA increases the performance of the test in comparison with the P. *falciparum *IFAT method.

However, no recombinant antigens from *P. ovale *and *P. malariae *are available up to now. Therefore, the sensitivity of the tests for *P. ovale *and *P. malariae *will probably be poor with assays using only antigens from two Plasmodium species. This is of importance especially for *P. malariae *infection, which is a frequent cause of transfusion malaria. Therefore, the sensitivity of the DiaMed ELISA malaria antibody test for *P. ovale *and *P. malariae *remains to be determined, as these two species were rare in the samples used here.

The DiaMed ELISA malaria test detects both malarial IgG and IgM antibodies because IgM antibody can be detected during malaria flare-ups and during both primary infection and re-infection, reflecting a response to new antigens (first encounter with *Plasmodium*, or first encounter with a new strain) [2,28]. The test uses also a crude *P. falciparum *antigen preparation to improve sensitivity. Indeed, *Plasmodium *epitopes are not uniformly recognised by all patients. Frequently exposed subjects show strong variability in their ability to mount a sustained antibody response to a given antigen [28,29]. However, use of anti-human IgM conjugates and of parasitic crude antigen may in theory predispose the assay to false positive results, but we found a satisfactory 99.6% specificity in non exposed to malaria blood donors.

Is ELISA able to replace IFAT in blood transfusion centres where serological routine screening is used [21]? For the moment, IFAT is still applied in Italy and Spain. If negative, donors are permitted to donate their blood [5]. In the UK, Hong Kong, and recently in France, malaria antibody ELISA is implemented, while it is under consideration in Australia [3]. These countries are applying these screening policies to detect malaria-risk donors, considering that transfusion-transmitted malaria is rare in non-endemic countries, with only three cases detected annually in the USA [30]. However, many blood donations are wasted because of the deferral or exclusion of donors who have recently travelled to endemic areas. When the DiaMed ELISA malaria antibody test kit was used to screen blood donors on a large scale, the results agreed with those of the IFAT reference method in 99.3% of cases of the not-exposed-to-malaria blood donors and in 96.8% in malaria-risk blood donors. The decreased specificity observed in malaria-risk blood donors compared to the not-exposed-to-malaria blood donors may be due to either false positive results or detection of antibodies directed to *P. vivax *antigens. Similar data were obtained by others with malaria ELISA using recombinant antigens [15]. However, the presence of *P. vivax *directed antibodies by *P. cynomolgi *IFAT in our samples was not confirmed. Numerous equivocal results were observed in the malaria risk blood donors, 1.5% for ELISA and 1.1% for IFAT, results considered in blood banks as positive following the precautionary principle. None of these equivocal samples were concordant with both methods. All these discordant samples and obviously the positive ones too are suspected to contain parasites. An Australian study was unable to detect any plasmodial antigen or DNA in 337 malaria-risk blood donors, positive or negative by a Newmarket malarial Ab ELISA, who had returned less than six months or more than six months from a malaria-endemic area [27]. In future, it would be of interest to investigate, by similar methods, the presence of Plasmodium sp. in all positive samples in the present study by ELISA and/or IFAT.

In summary, the DiaMed ELISA-malaria antibody test showed a better efficiency index than IFAT. That difference is essentially due to the better performance of this ELISA for the detection of *P. vivax *infected patients. The performance of this ELISA kit should also be extensively investigated to check if the discrepancies in results between ELISA and IFAT in samples from malaria-risk blood donors are indeed true cases of infection, especially *P. vivax *infections. A molecular approach is needed for that. The better reactivity observed with south-American patients and the 31 sera from donors with risk of malaria that tested negative by IFAT, but positive or equivocal by ELISA, may be due to cross reactivities with other protozoan parasites (Leishmania, Trypanosoma, Toxoplasma) [31]. An extensive expertise on sera from patients with these pathologies should also be considered. Anyway, overall efficiency and reliability of DiaMed ELISA malaria antibody test make it an interesting candidate for the replacement of IFAT in medical microbiology laboratories and in blood banks, where the need for an ELISA method that is easy to perform and suitable for automation is critical.

## Competing interests

DiaMed has provided the "DiaMed ELISA-Malaria antibody test" diagnostic kits and the financial support for the expertise of the reagents.

## Authors' contributions

EC, CD and AH designed the study and were in charge of performing the ELISA. PG and JPC provided the blood donor cases. YH provided the malaria cases. AS and TA contributed to the analysis of the cases. AWP performed the IFAT and all assays for malaria diagnosis.

## References

[B1] Greenwood B, Mutabingwa T (2002). Malaria in 2002. Nature.

[B2] Garraud O, Mahanty S, Perraut R (2003). Malaria-specific antibody subclasses in immune individuals: a key source of information for vaccine design. Trends Immunol.

[B3] Seed CR, Kitchen A, Davis TM (2005). The current status and potential role of laboratory testing to prevent transfusion-transmitted malaria. Transfusion Med Rev.

[B4] Kitchen A, Mijovic A, Hewitt PE (2006). Transfusion – transmitted malaria : current donor selection guidelines are not sufficient. Vox Sang.

[B5] Reesink HW (2005). European strategies against the parasite transfusion risk. Transfus Clin Biol.

[B6] Kitchen AD, Chiodini PL (2006). Malaria and blood transfusion. Vox Sang.

[B7] Leder K, Black J, O'Brien D, Greenwood Z, Kain KC, Schwartz E, Brown G, Torresi J (2004). Malaria in travelers : a review of the geosentinel surveillance network. Clin Infect Dis.

[B8] Slinger R, Giulivi A, Bodie-Collins M, Hindieh F, John RS, Sher G, Goldman M, Ricketts M, Kain KC (2001). Transfusion-transmitted malaria in Canada. Can Med Assoc J.

[B9] Candolfi E (2005). [Transfusion-transmitted malaria, preventive measures]. Transfus Clin Biol.

[B10] Coudert J, Garin J, Ambroise-Thomas P, Saliou P, Minjat M (1965). Utilisation de *Plasmodium cynomolgi bastinanellii *dans le sérodiagnostic par immuno-fluorescence des paludismes humains. Bull Soc Path Exot.

[B11] Wery M, Danis M, MouchetJ (1991). Diagnostic biologique. Paludisme.

[B12] Chiodini PL, Hartley S, Hewitt P, Barbara J, Lalloo K, Bligh J, Voller A (1997). Evaluation of a malaria antibody ELISA and its value in reducing potential wastage of red cell donations from blood donors exposed to malaria, with a note on a case of transfusion transmitted malaria. Vox Sang.

[B13] Contreras CE, Pance A, Marcano N, Gonzalez N, Bianco N (1999). Detection of specific antibodies to *Plasmodium falciparum *in blood bank donors from malaria-endemic and non-endemic areas of Venezuela. Am J Trop Med Hyg.

[B14] Silvie O, Thellier M, Rosenheim M, Datry A, Lavigne P, Danis M, Mazier D (2002). Potential value of *Plasmodium falciparum *-associated antigen and antibody detection for screening of blood donors to prevent transfusion-transmitted malaria. Transfusion.

[B15] Kitchen AD, Lowe PH, Lalloo K, Chiodini PL (2004). Evaluation of a malarial antibody assay for use in the screening of blood and tissue products for clinical use. Vox Sang.

[B16] Park CG, Chwae YJ, Kim JI, Lee JH, Hur GM, Jeon BH, Koh JS, Han JH, Lee SJ, Park JW, Kaslow DC, Strickman D, Roh CS (2000). Serologic responses of Korean soldiers serving in malaria-endemic areas during a recent outbreak of Plasmodium vivax. Am J Trop Med Hyg.

[B17] Ambroise-Thomas P (1976). Prévention du paludisme post-transfusionnel par sérodépistage des porteurs latents de Plasmodium parmi les donneurs de sang. Rev Fr Transfus Immunohematol.

[B18] Ambroise-Thomas P, Golvan Y, Ambroise-Thomas P (1984). Paludisme. Les nouvelles techniques en parasitologie.

[B19] Soler CP, Gerome P, Soullie B, Tissedre F, Bejan H, Yvetot J, Joussemet M (2003). Comparison of techniques used to screen blood donations for malaria antibodies. Med Trop (Mars).

[B20] Melancon-Kaplan J, Burns JM, Vaidya AB, Webster HK, Weidanz WP, Warren KS (1993). Malaria. Immunology and molecular biology of parasitic infections.

[B21] Garraud O, Relace J, Flori P, Perraut R (2004). Le risque de paludisme transfusionnel confronté à celui de la mutité biologique : deux données irréconciliables ?. Trans Clin Biol.

[B22] Mertens G, Vervoort T, Heylen S, Muylle L (1999). Malaria antibody ELISA insufficiently sensitive for blood donor screening. Vox Sang.

[B23] Spencer HC, Collins WE, Chin W, Skinner JC (1979). The enzyme-linked immunosorbent assay (ELISA) for malaria. I. The use of in vitro-cultured *Plasmodium falciparum *as antigen. Am J Trop Med Hyg.

[B24] Spencer HC, Collins WE, Skinner JC (1979). The enzyme-linked immunosorbent assay (ELISA) for malaria. II. Comparison with the malaria indirect fluorescent antibody test (IFA). Am J Trop Med Hyg.

[B25] Kim S, Ahn HJ, Kim TS, Nam HW (2003). ELISA detection of *P. vivax *malaria with recombinant multiple stage-specific antigens and its application to survey of residents in endemic areas. Korean J Parasitol.

[B26] Kim YM, Hwang HA, Yun WS, Kim SI, Lee KW, Park SK, Lee YJ, Kim TK, Wongsrichanalai C, Sakanari JA, Park H (2004). Efficacy of the merozoite surface protein 1 of *Plasmodium vivax *as an antigen for ELISA to diagnose malaria. Yonsei Med J.

[B27] Seed CR, Cheng A, Davis TM, Bolton WV, Keller AJ, Kitchen A, Cobain TJ (2005). The efficacy of a malarial antibody enzyme immunoassay for establishing the reinstatement status of blood donors potentially exposed to malaria. Vox Sang.

[B28] Perraut R, Guillotte M, Drame I, Diouf B, Molez J-F, Tall A, Trape J-F, Mercereau-Puijalon O, Spiegel A, Garraud O (2002). Evaluation of anti-*Plasmodium falciparum *antibodies in Senegalese adults using different types of crude extracts from various strains of parasite. Microbes Infect.

[B29] Aribot G, Rogier C, Sarthou J-L, Trape J-F, Balde AT, Druilhe P, Roussilhon C (1996). Pattern of immunoglobulin isotype response to *Plasmodium falciparum *blood-stage antigens in individuals living in a holoendemic area of Senegal (Dielmo, West Africa). Am J Trop Med Hyg.

[B30] Purdy E, Perry E, Gorlin J, Jensen K (2004). Transfusion-transmitted malaria: unpreventable by current donor exclusion guidelines?. Transfusion.

[B31] Abramo C, Fontes CJ, Krettli AU (1995). Cross-reactivity between antibodies in the sera of individuals with leishmaniasis, toxoplasmosis, and Chagas' disease and antigens of the blood-stage forms of Plasmodium falciparum determined by indirect immunofluorescence. Am J Trop Med Hyg.

